# Supplementary figures and images for: On the unfounded enthusiasm for soft selective sweeps II: Examining recent evidence from humans, flies, and viruses
Source: PLoS Genet. 2018 Dec 28;14(12):e1007859. doi: 10.1371/journal.pgen.1007859 (PMC6336318; doi:10.1371/journal.pgen.1007859)

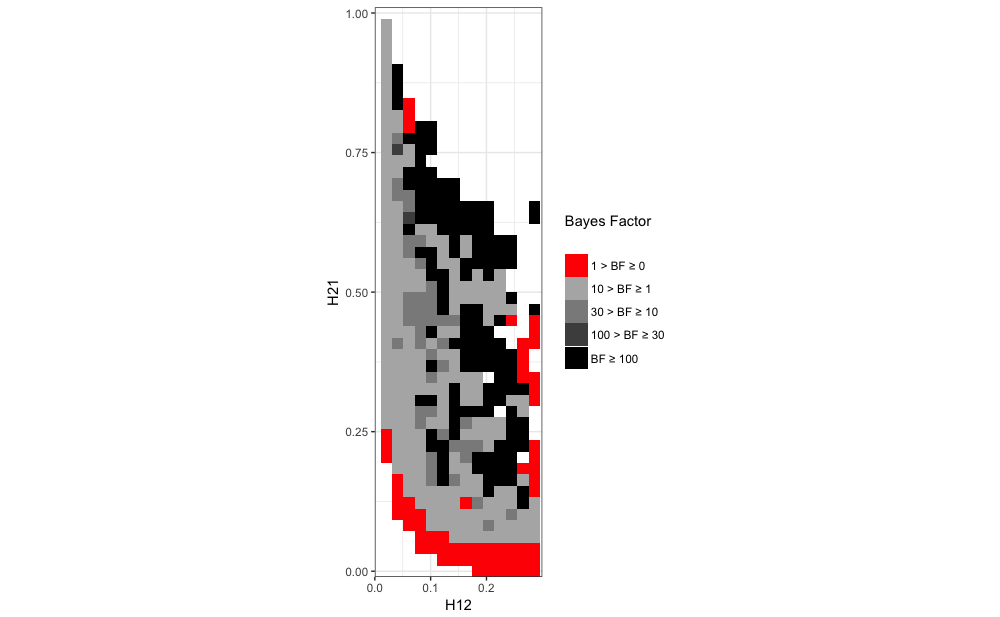

Supplement: S1 Fig — To demonstrate how the plotting of Bayes factors (BFs) can lead to erroneous conclusions about the ability of the H-statistics to differentiate between hard and soft sweeps, we here depict results in the same fashion as Garud et al. (their Fig 11). BFs were calculated by taking the ratio of the number of soft sweep versus hard sweep simulations that were within a Euclidean distance of 10% of a given pair of H12 and H2/H1 values. Red portions of the grid represent H12 and H2/H1 values that are more easily generated by hard sweeps (generated with Smu = 0.01), while grey portions represent regions of space more easily generated under soft sweeps (generated with Smu = 10). These results are based on 16,000 hard and soft sweep simulations. (TIFF) [file pgen.1007859.s001.tiff]

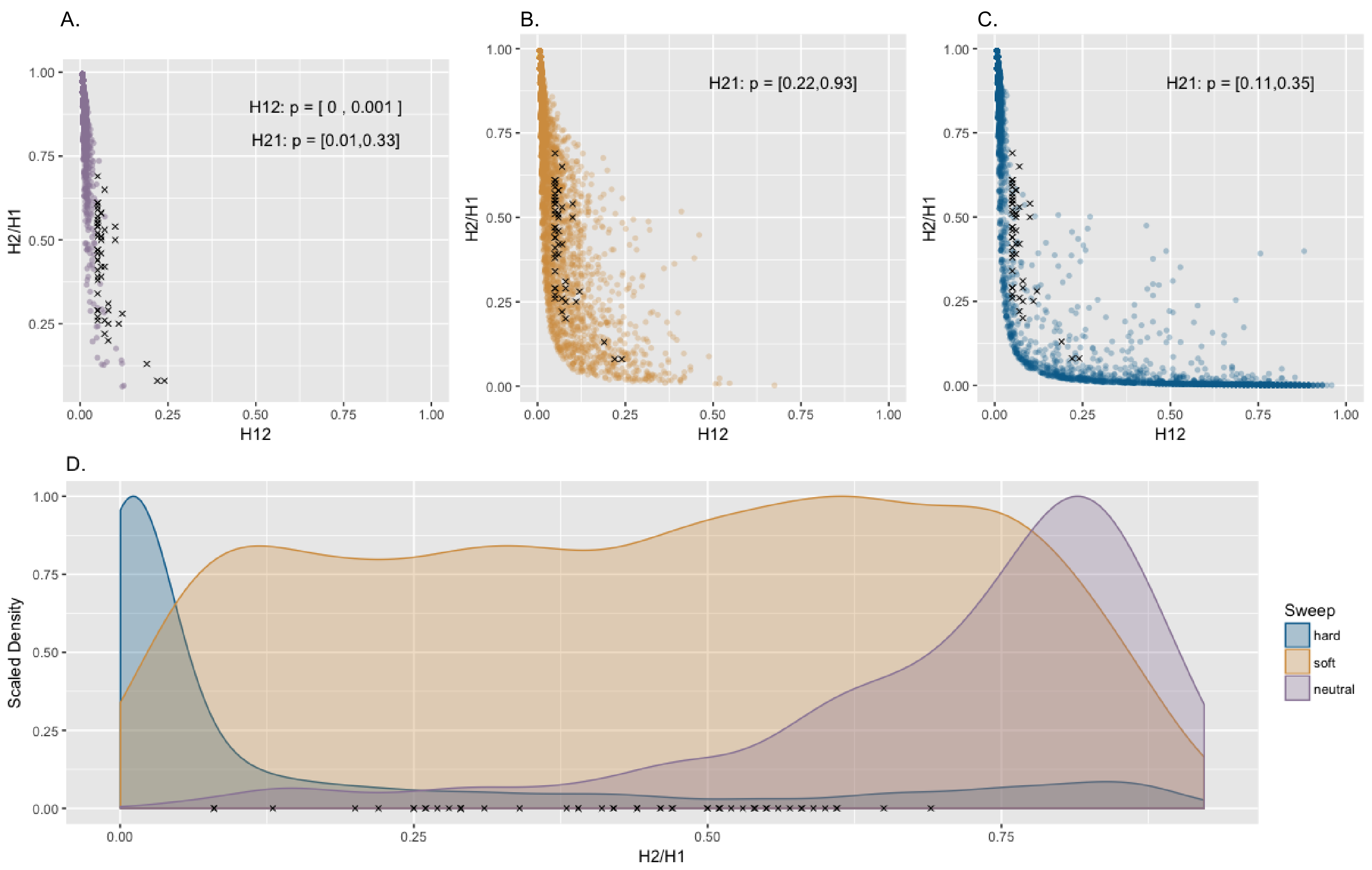

Supplement: S2 Fig — Distribution of H12 and H2/H1 values estimated under the 95% credibility interval of the DGRP admixture model of Duchen et al. [15] for (a) neutrality, (b) soft sweeps, and (c) hard sweeps. Simulations were conducted using windows of a fixed 400 SNPs. Additionally, all panels show the top 50 H12 outliers (black x's) from the empirical Drosophila data set that Garud et al. [10] concluded were soft sweeps. (d) Following their proposed practice, simulations generating the top 2.5% H12 values were ascertained from each set, and the scaled density of the corresponding H2/H1 values are plotted for these H12 outliers. As shown, this procedure artificially reduces the most extreme values observed under neutrality, resulting in three outlier regions remaining from amongst their initial 50. (TIFF) [file pgen.1007859.s002.tiff]

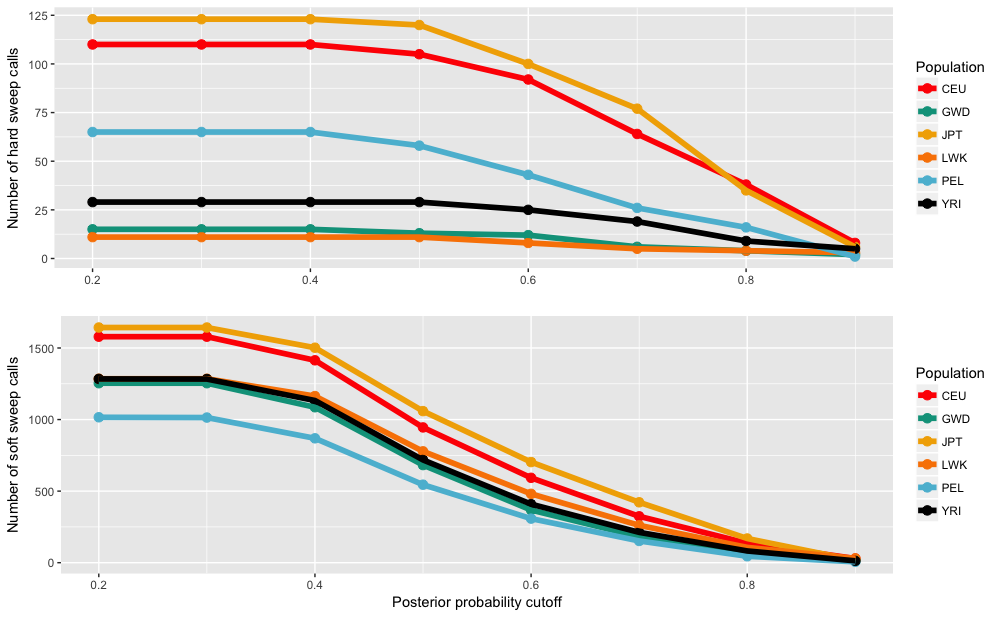

Supplement: S3 Fig — Drop-off in the number of genomic regions classified as hard (top) and soft (bottom) sweeps as a posterior probability threshold is imposed. Data plotted from Schrider and Kern’s S2 Table. (TIFF) [file pgen.1007859.s003.tiff]

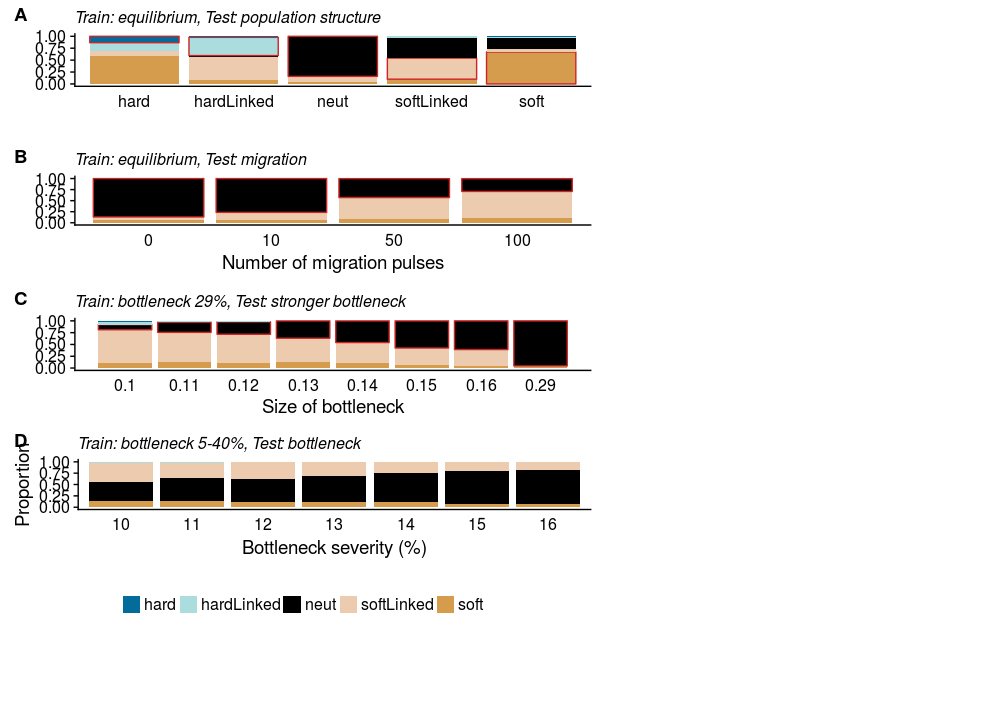

Supplement: S4 Fig — (a) Both test and training data were simulated under a constant size demographic model; however, the test data set consists of 100 individuals sampled from two populations (9:1 sampling ratio) which diverged 0.50 x 2N generations in the past. (b) The classification performance when neutrality is simulated under a structured, constant size population with migration, varying the number of pulses of gene flow. Here, the populations diverged 4N generations ago, with 10 migrants per pulse. (c) The classification performance of neutrality simulated under a bottleneck model with varying bottleneck severity (e.g., a 'size of bottleneck' of 0.1 corresponds to a temporary reduction to 10% of the ancestral size), when the true model is a bottleneck decreasing the population to 29% of the ancestral size. Bottleneck timing and duration are consistent across all test and training sets, with the bottleneck beginning at 0.044*4N generations ago and returning to the initial population size 0.0084*4N generations ago. (d) Similar to (c), however the training set was constructed from a range of bottleneck simulations which decrease the population to 5–40% of the ancestral size. (TIFF) [file pgen.1007859.s004.tiff]
