# Supplementary material for: On the unfounded enthusiasm for soft selective sweeps II: Examining recent evidence from humans, flies, and viruses
Source: PLoS Genet. 2018 Dec 28;14(12):e1007859. doi: 10.1371/journal.pgen.1007859 (PMC6336318; doi:10.1371/journal.pgen.1007859)
Supplement: S1 Text — msms commands used to simulate datasets for calculating H-statistics, discoal commands used to simulate data sets for S/HIC, and SLiM code used to simulate bottlenecked HIV populations. (DOCX) [file pgen.1007859.s007.docx]

***msms* commands**

The ms arguments for the DGRP model from Duchen *et al*. 2013 are:

msArguments = “totalSampleSize numberOfSimulations -t theta -I 3 sampleSizeAfrica sampleSizeEuro sampleSizeAmerica -n 2 scaledNeEuro -g 2 growthRateEuro -n 3 scaledNeAmerica -g 3 growthRateAmerica -es scaledTimeAdmix 3 proportionAdmix -ej scaledTimeAdmix 3 2 -ej scaledTimeAdmix 4 1 -ej scaledTimeSplitAfrEuro 2 1 -en scaledTimeAfricaExpansion 1 scaledNeAfricaBottleneck -en scaledTimeAfrCrash 1 scaledNeAfricaAncestral -r recombinationRate locusLength”

As in Garud *et al*, we set:

totalSampleSize = sampleSizeAmerica = 145

locusLength = 10000

𝜌 = 5*10^-7^

𝜇 = 10^-9^

sampleSizeAfrica = sampleSizeEuro = 0

All demographic parameters were sampled from the posterior distributions given in S1 Table (note that some of the variable names below correspond to the S1 Table column names). For use in the above msArguments, these were converted using the following equations:

scaledNeAfricaAncestral = Ne_anc

recombinationRate = 4 * Ne_anc * 𝜌 * locusLength

Theta = 4 * Ne_anc * 𝜇 * locusLength

scaledNeEuro = Ne_Eur / Ne_anc

scaledNeAmerica = Ne_Ame / Ne_anc

scaledTimeSplitAfrEurope = 10 ^log_time_split_AfrEur^ / (4 * Ne_anc)

scaledTimeAdmix = 10 ^log_time_admixture^ / (4 * Ne_anc)

EuroNe_anc = 10 ^log_Ne_Eur_bn^ / (4 * Ne_anc)

AmerNe_anc = 10 ^log_Ne_Ame_bn^ / (4 * Ne_anc)

AmerNe_cur = Ne_Ame / Ne_anc

growthRateEuro = -(1 / scaledTimeSplitAfrEuro) * ln(EuroNe_anc / scaledNeEuro)

growthRateAmerica = -(1 / scaledTimeAdmix) * ln(AmerNe_anc / AmerNe_cur)

scaledTimeAfrCrash = time_crash / (4 * Ne_anc)

scaledTimeAfrExpansion = scaledTimeAfrCrash - (1000 / (4 * Ne_anc))

scaledNeAfricaBottleneck = (1000 / 10^log_severity^) / Ne_anc

proportionAdmix = proportion_admixture

To implement selection, Garud *et al*. used msms.

./msms msArguments -Smu adaptiveMutationRate -SAA selectiveStrengthAA -Sa selectiveStrengthAa -SI startTime 3 0 0 FreqPop3 -Sp 0.5

Selection begins at startTime, which was sampled from ~*U*[0, scaledTimeAdmix]. Strength of selection and starting frequency of the beneficial mutation was scaled by the North American deme *N_e_* , where:

*N_e_* = scaledNeAmerica * *e*^-startTime * growthRateAmerica^ * Ne_anc

*s* ~ *U*[0,1]

selectiveStrengthAA = 2 * *N_e_ * s*

selectiveStrengthAa = *N_e_ * s*.

FreqPop3 = 1 / *N_e_*

For every set of randomly drawn demographic parameters and selection coefficients, we simulated 1) neutrality, 2) a hard sweep with an adaptiveMutationRate = 0.01, and 3) a soft sweeps with adaptiveMutationRate = 10.

***discoal* commands**

For equilibrium simulations: see Schrider and Kern’s (2016) S1 Table.

For LWK simulations: see Schrider and Kern’s (2017) S4 Table. We modified the selection strength so that -Pa 10 1000

For bottleneck simulations: see Schrider and Kern’s (2016) S1 Table under “Less severe bottleneck”. We tested a scenario where a sweep occurred 0.022 * 2*N* generations ago (-ws 0.022). We tested a range of bottleneck severities (0.05-0.40) by changing the argument -en 0.0084 0 <severity>.

For population structure simulations: we included the additional arguments to the equilibrium model -p 2 90 10 -ed <time since split> 0 1

For migration simulations: we included the additional arguments to the equilibrium model (here, we show three pulses of migration) -p 2 100 0 -ed <time since split> 0 1 -ea <pulse time 1> 0 1 0 <migration rate> -ea <pulse time 1> 0 1 0 <migration rate> -ea <pulse time 1> 0 1 0 <migration rate>

***SLiM* commands**

We initialize a population with the estimated mutation and recombination rates of HIV. The simulation requires us to specify values for :

's', the selection coefficient of beneficial mutations

'N', the population size

'bottleneck', the bottleneck size during treatment

'drms', the number of DRMs desired for the simulation run

'rarity', the number of generations between the introduction of new copies of each beneficial mutation

##################### START SLIM SCRIPT “softsweepslim.txt” #################

initialize() {

initializeMutationRate(3.4e-5);

initializeMutationType(1, 0.5, "f", 0);

// We initialize mutation types corresponding to the number of drms specified for this simulation run.

for (number in c(2:(drms + 1))) {

initializeMutationType(number, 0.5, 'f', s);

}

initializeGenomicElementType("g1", m1, 1.0);

initializeGenomicElement(g1, 0, 1980);

initializeRecombinationRate(1.4e-5);

// We create a vector of generations in which to introduce beneficial mutations, after 1000 generations of neutral evolution. These generations occur with a frequency determined by the 'rarity' variable.

x = c(0:100000);

x = x * rarity;

x = x + 1000;

defineConstant("mutgens",x);

// We select random sites at which to introduce these beneficial mutations for each DRM

defineConstant('sites', sample(c(0:1979), drms));

}

1 {

sim.tag = 0;

sim.addSubpop("p1", 1);

p1.tag = 0;

countn = 0;

ntrial = 1;

while (ntrial < N){

countn = countn + 1;

ntrial = ceil(ntrial * 1.1);

}

defineConstant('countn2', countn);

defineConstant('countn3', 0);

for (gen in mutgens){

sim.registerLateEvent(NULL, s1.source, gen, gen);

}

sim.deregisterScriptBlock(s1);

}

// The population grows exponentially until reaching a size of N, then evolves neutrally for a total of 1000 generations.

s1 2 late() {

if (sum(p1.individuals.countOfMutationsOfType(2)) == 0 & (sum(sim.substitutions.mutationType == m2) < 1)){

p1.genomes[0].addNewDrawnMutation(2, sites[0]);

}

if (drms > 1) {

for (number in c(3:(drms + 1))){

if ((sum(sim.substitutions.mutationType.id == number) < 1) & (sum(sim.substitutions.mutationType.id == (number - 1)) == 1) & (sum(p1.individuals.countOfMutationsOfType(number)) == 0)) {

p1.genomes[0].addNewDrawnMutation(number, sites[number - 2]);

}

}

}

}

2:998 {

currentnumb = countn3;

if (currentnumb < countn2){

p1.setSubpopulationSize(asInteger(ceil(p1.individualCount * 1.1)));

}

if (currentnumb == countn2){

p1.setSubpopulationSize(N);

}

rm('countn3', removeConstants = T);

defineConstant('countn3', currentnumb + 1);

}

999 {

p1.setSubpopulationSize(bottleneck);

}

// After 1000 generations, treatment is induced, and the population is bottlenecked. Every set number of generations, according to the 'rarity' variable, we check if a beneficial mutation is segregating in the population. If it is not, we introduce a single copy of the mutation. As each DRM is fixed, the beneficial mutations introduced belong to a unique mutation class, so that the total number of DRMs can be counted. Each generation, we check how many total DRMs have fixed. When we reach the desired number of DRMs for this simulation, the population recovers to a size of 2*N genomes and evolves neutrally for 100 generations, before outputting the entire population state, from which we calculate the number of expected ambiguous sites. Since we only introduce a new copy of a mutation when the previous DRM has fixed or the current DRM has been lost, we only permit hard selective sweeps. After each successful sweep, the population recovers 20% of the bottleneck size, reflecting an increase in the total fitness of the population.

1001: late() {

countingfinal = 0;

for (number in c(2:(drms + 1))){

countingfinal = countingfinal + sum(sim.substitutions.mutationType.id == number);

}

if (sim.tag == 0){

p1.setSubpopulationSize(asInteger(countingfinal * (2 * bottleneck) + bottleneck));

}

if (countingfinal == drms & sim.tag == 0) {

p1.tag = sim.generation + 100;

p1.setSubpopulationSize(N);

sim.tag = 1;

print('fixation of all drms achieved');

}

if (sim.tag == 1) {

if (sim.generation == p1.tag){

sim.outputMutations(sim.mutationsOfType(m1));

sim.simulationFinished();

}

}

}

###################### END SLIM SCRIPT “softsweepslim.txt” ##################

This SLiM code functions similarly to the softsweepslim.txt code, with the difference that here we do not introduce beneficial mutations. This code was used to measure the number of ambiguous base calls in populations that experience the same growth and bottleneck conditions as the other simulations without the introduction and fixation of DRMs, *i.e*., populations that do not evolve resistance.

################### START SLIM SCRIPT “softsweepslim0drms.txt” ###############

initialize() {

initializeMutationRate(3.4e-5);

initializeMutationType(1, 0.5, "f", 0);

initializeGenomicElementType("g1", m1, 1.0);

initializeGenomicElement(g1, 0, 1320);

initializeRecombinationRate(1.4e-5);

}

1 {

sim.tag = 0;

sim.addSubpop("p1", 1);

p1.tag = 0;

countn = 0;

ntrial = 1;

while(ntrial<N) {

countn = countn+1;

ntrial = ceil(ntrial*1.1);

}

defineConstant('countn2', countn);

defineConstant('countn3', 0);

bottlenecker = bottleneck * 2 * 0.95;

defineConstant("bottleneck2", bottlenecker);

}

2:998 {

currentnumb = countn3;

if (currentnumb < countn2) {

p1.setSubpopulationSize(asInteger(ceil(p1.individualCount * 1.1)))

}

if (currentnumb==countn2) {

p1.setSubpopulationSize(N);

}

rm('countn3',removeConstants = T);

defineConstant('countn3', currentnumb + 1);

}

999 {

p1.setSubpopulationSize(bottleneck);

}

2000 {

p1.setSubpopulationSize(5000);

}

2100 late() {

sim.outputMutations(sim.mutationsOfType(m1));

sim.simulationFinished();

}

#################### END SLIM SCRIPT “softsweepslim0drms.txt” ################
